# Central Texas Medical Association

**Published:** 1900-06

**Authors:** A. C. Scott, N. A. Olive

**Affiliations:** President; Waco, Texas; Secretary; Waco, Texas


### Central Texas Medical Association.

WACO, TEXAS, June 4, 1900.

*Dear Doctor:*

Your active interest in progressive medicine and participation in society work in the past warrants the belief that you will contribute something of interest to the July meeting of the Central Texas Medical Association. It was the opinion of old members that the January meeting surpassed, in interest, any previous assembly of our society. Can you not lend us your enthusiastic support and present a paper on some interesting subject, or report one or a series of cases, the discussion of which may prove benefiicial to all present?

The general practitioner of medicine is less able to spare the time from his society work than from his practice, and his patients can better afford to spare him a few days every year than tolerate the deficiencies incurred by non-association with his fellow-practitioners in the interesting discussion of subjects of vital importance to all.

Following is a list of section chairmen. Please send in subject of your paper as early as possible:

*Section of General Medicine*—Dr. J. W. Embree, Belton, Chairman.

*Section of Surgery*—Dr. W. C. Blalock, Kosse, Chairman.

*Section of Obstetrics and Gynecology*—Dr. A. R. Kuykendall, Morgan, Chairman.

*Section of Diseases of Nervous System and Medical Jurisprudence*—Dr. Jno. S. Turner, Terrell, Chairman.

The society will meet in Waco the second Tuesday and Wednesday in July, 1900.

Yours fraternally,

A. C. SCOTT, President. N. A. OLIVE, Secretary.

